# Identification of a highly drought-resistant *pp7l hda6* mutant

**DOI:** 10.3389/fpls.2024.1341576

**Published:** 2024-06-03

**Authors:** Duorong Xu, Dario Leister, Tatjana Kleine

**Affiliations:** Plant Molecular Biology, Faculty of Biology, Ludwig-Maximilians-University Munich, Planegg-Martinsried, Germany

**Keywords:** cell wall, chloroplast, drought, histone deacetylase, PROTEIN PHOSPHATASE7-LIKE (PP7L), (post)transcriptome

## Abstract

Plants have developed efficient strategies to counteract drought stress, including stomata closure, significant changes in nuclear gene expression, and epigenetic mechanisms. Previously, we identified *Arabidopsis thaliana* PROTEIN PHOSPHATASE7-LIKE (PP7L) as an extrachloroplastic protein that promotes chloroplast development. In addition, it was shown that PP7L is involved in high light and salt tolerance. Here, we demonstrate that the *pp7l* mutant can withstand prolonged periods of drought stress. Interestingly, despite impaired growth under standard growth conditions, photosynthetic efficiency recovers in *pp7l* mutant plants experiencing drought conditions. To assess the (post)transcriptional changes occurring in the *pp7l* mutant under different durations of drought exposure, we used an RNA-sequencing technique that allows the simultaneous detection of organellar and nuclear transcripts. Compared with the previously reported drought-responsive changes in the wild type, the drought-responsive changes in organellar and nuclear transcripts detected in the *pp7l* mutant were negligible. Our analysis of the data generated in this study and review and analysis of previous literature motivated us to create a *pp7l hda6* (*histone deacetylase 6*) mutant, which exhibits remarkable drought resistance. Notably, the growth penalty associated with *pp7l* was alleviated in the double mutant, ruling out a dwarf effect on the drought-tolerant trait of this genotype. Future studies may consider that multiple loci and factors are involved in stress resistance and explore combinations of these factors to create even more resilient plants.

## 1 Introduction

Abiotic stress is one of the major constraints to global crop production and food security. Drought is caused not only by low precipitation, but also by temperature dynamics and light intensity, making it a key abiotic stress (Singh et al., 2018). Drought affects morphological, physiological, biochemical, and molecular characteristics of plants, with negative effects on photosynthetic capacity (Seleiman et al., 2021). Physiological changes may include the closure of stomata, changes in cell wall integrity, increased water use efficiency (WUE), and a reduction in relative water content (RWC), while biochemical changes include for example higher phytohormone production, in particular abscisic acid (ABA), and declined photosynthesis (Seleiman et al., 2021). However, a response to drought can have both positive and negative effects. Moreover, it is essential to consider that plant responses to drought involve a complex interplay of multiple factors, and understanding is far from complete. For example, stomatal closure prevents water loss by reducing transpiration, but also reduces the rate of photosynthesis by reducing CO_2_ uptake (Medrano et al., 2002; Lawson and Blatt, 2014).

At the molecular level, the response to drought and other stresses is accompanied by large-scale changes in nuclear (e.g., Bashir et al., 2019; Bao et al., 2020; Mahmud et al., 2022), and plastid (Xu et al., 2023) gene expression. In addition, in recent years, histone modifications have been shown to be among the epigenetic mechanisms that control gene expression in response to abiotic stress (Luo et al., 2017; Nunez-Vazquez et al., 2022), in particular drought (Li et al., 2021), by altering chromatin accessibility. One of the modifications to histones involves histone acetylation, which adds acetyl groups to histone proteins through the action of histone deacetylases (HDACs). Histone acetylation is usually linked to a relaxed chromatin structure, which allows genes to be more accessible for transcription. The HDACs that have been most thoroughly researched in *Arabidopsis thaliana* (hereafter, Arabidopsis) are HDA6 and HDA19. These HDACs are essential players in pathogen defense, regulation of flowering, senescence, and responses to abiotic stress (Nunez-Vazquez et al., 2022; Cui et al., 2023). Interestingly, it is not clear whether HDA6 and HDA19 have antagonistic functions in the regulation of salt stress. For example, based on germination efficiency, *hda6* and *hda19* mutants are more sensitive to ABA and salt stress (Chen et al., 2010; Chen and Wu, 2010). But based on the determination of chlorophyll degradation during drought, deficiency in HDA19 leads to increased salt tolerance (Mehdi et al., 2016), and Ueda et al. (2017) also demonstrated that the *hda19* mutant is salt tolerant. The latter result was based on the assessment of survival under salt stress. However, it is clear that both *hda6* (Kim et al., 2017) and *hda19* (Ueda et al., 2018) mutants show improved drought tolerance.

We previously studied PROTEIN PHOSPHATASE7-LIKE (PP7L) and showed that *pp7l* mutants also exhibit decreased salt tolerance (Xu et al., 2020b). Interestingly, PP7L promotes chloroplast development (Xu et al., 2019), although it is localized to the nucleus and cytosol and not to chloroplasts (Xu et al., 2019; de Luxán-Hernández et al., 2020). Although PP7L is a member of a small subfamily of phosphoprotein phosphatases (PPPs), it does not act as a phosphatase, because it lacks the conserved amino acids required for a catalytic center (Farkas et al., 2007; de Luxán-Hernández et al., 2020), and has therefore been termed “inactive PP7” (Farkas et al., 2007). PP7L interacts with MAINTENANCE OF MERISTEMS (MAIN) and MAIL1, which encode plant mobile domain proteins essential for mRNA expression of a subset of protein-coding genes, transposable elements (TE) silencing, and primary root development (de Luxán-Hernández et al., 2020; Nicolau et al., 2020). Mutants lacking PP7L display developmental phenotypes similar to those of the *main* or *mail1* mutants, suggesting that PP7L, MAIN and MAIL1 act in the same pathway. Indeed, in addition to being involved in the expression of a substantial number of protein-coding transcripts (Xu et al., 2019), PP7L has also been implicated in the silencing of TEs (de Luxán-Hernández et al., 2020; Nicolau et al., 2020).

Loss of PP7L results in pleiotropic phenotypes, including reduced resistance to high light and temperature stress (Xu et al., 2019; Xu et al., 2020b), although the underlying molecular mechanism is unclear. However, the involvement of PP7L in the silencing of TEs (de Luxán-Hernández et al., 2020; Nicolau et al., 2020) and the altered resistance of histone deacetylase mutants to stress reinforce the notion that stress resistance is closely linked to different types of epigenetic processes.

In this study, we found that the *pp7l* mutant also exhibits remarkable drought tolerance. Furthermore, photosynthesis in *pp7l* recovers during drought stress, albeit the mutant exhibits reduced photosynthetic performance under well-watered growth conditions. The drought-tolerance phenotype of *pp7l*, together with PP7L’s function in high light and temperature acclimation, makes PP7L an interesting candidate for an integrator of multiple stresses. To learn more about the underlying molecular phenotype, we investigated the transcriptional behavior of the *pp7l* mutant under drought. We integrated our RNA-Seq analysis with previously published wild-type RNA-Seq (Xu et al., 2023) and microarray (Kim et al., 2017) data, which motivated us to re-investigate drought tolerance of cell wall mutants, and to introduce a mutation in the histone deacetylase gene *HDA6* into *pp7l*. Strikingly, this led to the identification of an extremely drought-resistant *pp7l hda6* mutant in which the growth retardation seen in *pp7l* plants is relieved.

## 2 Materials and methods

### 2.1 Plant material and growth conditions

The *Arabidopsis thaliana* lines implemented in this investigation are in the Columbia (Col-0) genetic background. The mutants analyzed in this study included *pp7l-1*, *pp7l-2*, *pp7l-3*, *cry1cry2*, *cop1–4*, which were used previously (Xu et al., 2019), *hda6–7* (N66154; also known as *rts1–1*) (Aufsatz et al., 2002) and *sal1* (SALK_020882) (Wilson et al., 2009). The T-DNA line SALK_201895 (*hda6–11*) was obtained from the Arabidopsis Biological Resource Center. The homozygous *hda6–11*, *pp7l-1 hda6–11* and *pp7l-2 hda6–11* mutant lines were identified using primers suggested by the SALK Institute (http://signal.salk.edu/cgi-bin/tdnaexpress). The position of the T-DNA insertion in *HDA6* was confirmed by sequencing and lies +4 bp from the start codon of the *AT5G63110* gene.

Seeds were sown onto 1/2 MS medium that consisted of 1% (w/v) sucrose and 0.8% (w/v) agar, followed by incubation at 4°C in darkness for two days. Thereafter plates were transferred to a climate chamber under a 16-h-light and 8-h-dark cycle, where the light intensity was maintained at 100 µE m^-2^ s^-1^ with a temperature of 22°C. For the drought treatments, seven-day-old seedlings were transplanted into pots (potting soil A210, Stender, Schermbeck, Germany) and grown for additional two weeks under standard growth conditions consisting of a 16-/8-h light/dark cycle (illumination was provided from HQI Powerstar 400W/NDL lamps, with a fluence of ∼90 µmol photons m^−2^ s^−1^ on leaf surfaces). In addition, temperature (22°C/20°C during the day/night cycle) and relative humidity (60%) were strictly controlled under all conditions. Three-week-old plants either continued to grow under standard growth conditions or were exposed to drought conditions by not being watered for the specified periods. A camera (Canon 550D, Krefeld, Germany) was used to document the drought phenotypes.

**Figure 1:**
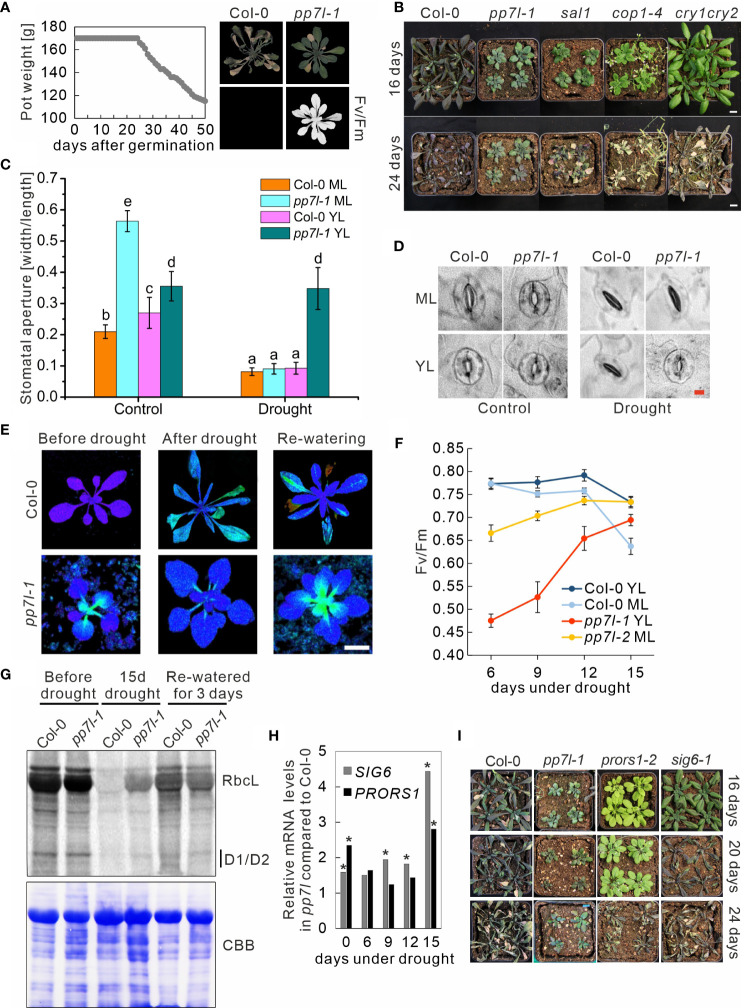
Soil-grown *pp7l* plants exhibit higher drought tolerance compared to the wild type. **(A)** The experimental design involved controlling water status by maintaining consistent pot weight. Gradual reduction of pot weight was carried out after 24 days. While detectable photosynthetic activity (maximum quantum yield of photosystem II, Fv/Fm) indicated continued viability of *pp7l* plants, 46-day-old WT (Col-0) plants failed to survive under similar conditions. **(B)** WT and the various genotypes were cultivated for three weeks under standard conditions, followed by withholding water for 16 and 24 days. Bar = 1 cm. **(C)** Analysis of stomatal apertures of WT and *pp7l-1* plants treated as in panel **(B)** Each column represents the mean value of data for three biological replicates. Bars show standard deviations and lowercase letters (a to e) indicate independent classes according to Student’s t-test with a significance level of *P* < 0.001. **(D)** Illustrative images of stomata from WT and the *pp7l-1* mutant were captured under well-watered conditions and after experiencing drought for 12 days. Bar = 10 μm. **(E)** Recovery of chloroplast development in *pp7l* plants following drought stress documented by Imaging-PAM pictures of 3-week-old *pp7l* plants taken before and after exposure to drought stress induced by withholding water for 15 days (15d drought), and after re-watering for 3 days. **(F)** The graph presents Fv/Fm values of 3-week-old WT and *pp7l* mutant plants exposed to drought stress for the specified time period. The data illustrate mean values ± SDs of three independent experiments, each with eight plants. YL indicates young leaves, and ML indicates mature leaves. **(G)** *In vivo* pulse labeling of chloroplast proteins with [^35^S]Met in the presence of cycloheximide shows that translation takes place at a higher rate in *pp7l-1* chloroplasts under drought stress than under conditions of re-watering. After pulse-labeling for 30 minutes, proteins were separated by SDS-PAGE and viewed via autoradiography. The Coomassie-Brilliant-Blue (CBB)-stained membrane served as a loading control. **(H)** Fold changes of *SIG6* and *PRORS1* mRNA levels under control conditions and during drought treatment in *pp7l* relative to WT plants. Data were extracted from RNA-Seq results. Asterisks indicate data with an adjusted *P*-value of < 0.01. **(I)** WT and mutant plants were cultured for three weeks under standard growth conditions before being deprived of water for durations of 16, 20, and 24 days.

For the tightly controlled drought experiments shown in **Figure 1A**, plants were grown under short-day conditions (10 h light of 115 μE m^−2^ s^−1^, 22°C, 45% relative humidity/14h darkness, 20°C, 60% relative humidity). Plant growth and watering were controlled by a conveyor belt system (Photon Systems Instruments, Ltd., Drasov, Czech Republic) essentially as described by Xu et al. (2023). Briefly, seeds were stratified for 3 days, and then sown directly onto soil. After 5 days, the seedlings were transferred to pots. Each pot contained 100 g of moist soil and sand mixture (150 ± 0.5 g total pot weight), and genotypes were placed in randomized positions. Pot weight was adjusted to 165 g within two days by multiple watering. 18-day-old plants were subjected to drought treatment, during which the pots lost approximately 3 g/day until pot weight reached 110 g (age of the plants: 39 days). Pots were weighed daily and watered automatically to ensure consistent weight loss for each pot. When the Fv/Fm (maximum quantum yield of photosystem II) value for wild-type plants was close to zero, drought treatment was terminated by gradually re-watering the pots to 130 g over a week to allow recovery, and surviving plants were monitored by determination of the Fv/Fm value.

### 2.2 Stomatal aperture measurement

For stomatal aperture measurements epidermal strips were used. After being kept in the dark for 24 h, the stomata of *pp7l* plants were significantly open (see **Supplementary Figure 1**). Thus, all of the plants were initially kept in darkness for 48 h, and were then illuminated with 30 µmol m^-2^ s^-1^ blue light, 50 µmol m^-2^ s^-1^ red light or 50 µmol m^-2^ s^-1^ far-red light for 2 days. Epidermal strips of leaves were peeled off the abaxial side of the leaf under dim light using forceps. To examine drought-mediated stomatal closure, epidermal strips were peeled from plants growing under control or drought conditions. Stomatal apertures were photographed under a microscope (Zeiss, Oberkochen, Germany) and measured with the help of ImageJ software.

### 2.3 Chlorophyll *a* fluorescence measurement

Chlorophyll fluorescence parameters were measured using an imaging Chl fluorometer (Imaging PAM, M-Series; Walz, Effeltrich, Germany). The method employed involved dark adaptation for 30-min, followed by the determination of Fv/Fm. F_0_ was measured at a low frequency of pulse-modulated measuring light (4 Hz, intensity 3, gain 3, damping 2), while Fm was quantified following saturation pulses of approximately 2700 µmol photons m^−2^ s^−1^ for 0.8 s. The parameters were calculated and plotted with the ImagingWinGigE software.

### 2.4 *In vivo* labeling of chloroplast proteins

Chloroplast protein labeling was performed essentially as described previously (Xu et al., 2019) with the following modifications. For *in vivo* labeling, 100 mg of young leaves were incubated in the labeling buffer for 30 minutes. The labeling buffer contained 10 mM Tris-HCl, 5 mM MgCl_2,_ 20 μg/mL cycloheximide, 20 mM KCl, pH 6.8, and 0.1% (v/v) Tween 20, to impede the synthesis of nuclear-encoded proteins. Subsequently, [^35^S]Met (1 mCi) was added to the same solution. After labeling for 30 min under light conditions of 80 μmol photons m^−2^ s^−1^, the leaves were washed and frozen in liquid nitrogen. Subsequently, total proteins were isolated and subjected to SDS-PAGE. Radiolabeled proteins were visualized by autoradiography (Typhoon™ laser scanner, GE Amersham).

### 2.5 Nucleic acid extraction

100 mg of leaf tissue was homogenized in extraction buffer containing 200 mM Tris/HCl (pH 7.5), 25 mM NaCl, 25 mM EDTA and 0.5% (w/v) SDS. Following centrifugation, DNA was precipitated from the supernatant by the addition of isopropyl alcohol. The DNA was then washed with 70% (v/v) ethanol and dissolved in distilled water.

### 2.6 RNA-sequencing

Total RNA was isolated utilizing TRIzol Reagent™ (Thermo Fisher Scientific, Waltham, MA, USA) and subsequently purified using the RNA Clean & Concentrator kit (Zymo Research, Irvine, USA), following the manufacturer’s instructions. The quality and integrity of the RNA was evaluated with an Agilent 2100 Bioanalyzer (Santa Clara, USA). Ribosomal RNA depletion, RNA-Seq library generation, and paired-end (150 bp) sequencing were performed on an Illumina HiSeq 2500 system (Illumina, San Diego, USA) following the protocol outlined by Xu et al. (2023). Each condition (0, 6, 9, 12 and 15 days) was assessed using three independent biological replicates. The sequencing data have been deposited to Gene Expression Omnibus (Edgar et al., 2002) under the accession number GSE202931.

### 2.7 Chloro-Seq and 3D RNA-Seq analysis

To detect editing and splicing efficiencies of chloroplast-encoded transcripts, a modified Chloro-Seq pipeline (Malbert et al., 2018) was used as described (Xu et al., 2023).

To determine overall transcript accumulation, we implemented the 3D RNA-Seq pipeline (Guo et al., 2021). For this purpose, RNA-Seq reads were prepared using the Galaxy platform (https://usegalaxy.org/). After adaptor removal with Trimmomatic (Bolger et al., 2014), sequencing quality was tested with FastQC (http://www.bioinformatics.babraham.ac.uk/projects/fastqc/). Transcript abundances were then calculated using Salmon (Patro et al., 2017), with AtRTD2-QUASI serving as a reference transcriptome (Zhang et al., 2017). The generated files were then uploaded to the 3D RNA-Seq app (https://3drnaseq.hutton.ac.uk/app_direct/3DRNAseq; Calixto et al., 2018; Guo et al., 2021) to calculate transcript per million reads (TPMs) with the implemented lengthScaledTPM method. Lowly expressed transcripts and genes were removed based on the data’s mean-variance trend. The relative expression values were computed in accordance with Xu et al. (2023).

### 2.8 cDNA synthesis and quantitative reverse transcriptase-polymerase chain reaction analysis

cDNA synthesis and quantitative real-time PCR analysis were performed essentially as described (Xu et al., 2023) with minor modifications which included the usage of the HiScript^®^ III RT SuperMix for qPCR (+gDNA wiper) kit (Nanjing Vazyme Biotech, Nanjing, PRC). The following primer pair was used for the specific detection of *ALDH2B7* mRNAs: forward primer (spanning exon 1 and exon 2), 5′-TCTATCTGCTCTTCTTGTTGGG-3′; reverse primer (binding to exon 3), 5′-GAAAGCAACCTTATCAACGTCCA-3′. Results were normalized to mRNA expression levels of *AT3G58500* encoding PROTEIN PHOSPHATASE 2A-4: forward primer, 5′- CAGACCTCTTTGACTATTTCCCA-3′; reverse primer, 5′- TTGTTCCGAAATATCCTGACCA-3′.

### 2.9 Determination of Gene Ontology enrichments

The Database for Annotation, Visualization and Integrated Discovery (DAVID; Huang da et al., 2009) was utilized to calculate GO enrichments. A 5-fold enrichment cut-off was applied, compared to the expected Arabidopsis genome frequency, along with an FDR (Benjamini-Hochberg) ≤ 0.05.

## 3 Results and discussion

### 3.1 The *pp7l* mutant can survive long periods of drought stress – also under strictly controlled water conditions

During further phenotypical characterizations of the *pp7l* mutant, we observed that the stomata of *pp7l* mutant seedlings were constitutively open under blue, red, and far-red light, as well as in darkness (**Supplementary Figure 1A**), and the width-to-length ratio of the stomatal aperture was significantly higher under all conditions (**Supplementary Figure 1B**). This is reminiscent of the stomatal behavior of the *cop1–4* (*constitutively photomorphogenic1–4*) mutant (Mao et al., 2005). Moreover, when ABA (abscisic acid) was applied to isolated *cop1–4* leaves, as well as when *cop1–4* plants were grown on soil and then exposed to drought conditions, they do not close their stomata. Accordingly, water loss from detached *cop1–4* leaves is higher than from wild-type leaves. Nevertheless, soil-grown *cop1–4* copes better with drought than the wild type (Moazzam-Jazi et al., 2018). To test whether *pp7l* mutants also displayed altered tolerance to drought, water was deprived from 3-week-old wild-type (WT; Col-0) and *pp7l-1* mutant plants for 18 days. Interestingly, upon re-watering of these plants for three days, 60% of them still were healthy, whereas all WT plants died (**Supplementary Figure 1C**). In addition, when water was withheld for 24 days, again all WT plants died, while all *pp7l-1* plants survived (**Supplementary Figure 1D**). Notably, the drought tolerance phenotype was also observed with *pp7l-2* and *pp7l-3* plants (**Supplementary Figure 1E**). However, *pp7l* is a dwarf mutant (Xu et al., 2019). Thus, it is possible that *pp7l* and other dwarf mutants are more resistant to drought conditions because their roots extract water from the soil at a lower rate. To further investigate this, an alternative drought experiment was conducted, subjecting both wild-type and *pp7l-1* to identical drought conditions by manipulating pot weights through controlled watering as described in the Materials and Methods section (see **Figure 1A**). Because the measurement of chlorophyll fluorescence is a quick and noninvasive way to evaluate how drought stress affects the performance of photosystem II (PSII) (Chen et al., 2016; Hu et al., 2023), phenotypes and Fv/Fm (maximum quantum yield of PSII) values were determined. Remarkably, the control WT plants perished after 46 days of drought treatment, whereas the *pp7l-1* mutant still maintained photosynthetic activity as indicated by the measurement of Fv/Fm (**Figure 1A**). Notably, this experiment was conducted under short-day conditions, showing that the drought resistance of the *pp7l* mutant is independent of the day length. In another experimental set-up, we included the dwarf mutants *cop1* (higher water loss under drought; Moazzam-Jazi et al., 2018) and *sal1* (no higher water loss of detached rosettes under drought; Wilson et al., 2009) as controls. Because it is likely that COP1 acts as a repressor of stomatal opening downstream of the cryptochrome (CRY) pathway, and the non-dwarf *cry1 cry2* mutant is also drought tolerant (Mao et al., 2005), this double mutant was also included in our study. Photographs were taken after 16 and 24 days of withholding water from 3-week-old plants. All mutants appeared to be drought-tolerant after 16 days. They accumulated less anthocyanin and leaves looked healthier than those of the WT (**Figure 1B**). The remarkable ability of the *pp7l* mutant to cope with drought stress was again underpinned after 24 days. After this period, *pp7l* showed no signs of wilting, while *sal1* had begun to wilt, and *cop1–4* and *cry1 cry2* were clearly wilting. Analysis of epidermal stomata from the abaxial side of mature and young WT and *pp7l* leaves showed that *pp7l* stomata had a higher width-to-length ratio than WT under standard growth conditions (**Figures 1C, D**). Strikingly, mature leaves of *pp7l* were able to close their stomata just as well as mature and young leaves of the WT even under drought exposure. However, stomata of young *pp7l* leaves were indifferent to drought stress and showed the same open appearance as under control conditions (**Figures 1C, D**).

In conclusion, the hypothesis of differential water usage by the dwarf mutants cannot be dismissed, however we can classify *pp7l* as a bona fide survivor of long drought periods.

### 3.2 Photosynthetic efficiency recovers in *pp7l* plants during drought stress

Chloroplast development in young leaves is delayed in plants lacking functional PP7L under standard growth conditions (Xu et al., 2019; **Figure 1E**), and the difference in stomatal behavior between young and mature *pp7l* leaves (see **Figures 1C, D**), and the still green leaves of the *pp7l* mutant under drought stress (**Figures 1A, B**; **Supplementary Figure 1**) are noteworthy. To determine whether photosynthetic performance was also differentially regulated during drought, Fv/Fm was measured in young and mature leaves after 6, 9, 12 and 15 days of drought stress. Fv/Fm of WT leaves remained relatively constant (at around 0.77) for up to 12 days of drought exposure; after 15 days it had dropped to 0.74 and 0.63 in young and mature leaves, respectively (**Figure 1F**). Surprisingly, but in agreement with the healthier appearance of *pp7l*, the low Fv/Fm values measured in *pp7l* leaves (0.47 and 0.66 for young and mature leaves, respectively) rose during the drought-stress period to 0.69 and 0.74, respectively, showing a pronounced recovery (especially of young leaves) after 15 days of drought exposure (**Figures 1E, F**). During the re-watering period, Fv/Fm values of *pp7l* leaves resembled the low values observed for *pp7l* plants grown under continuous, well-watered conditions. Previously it was shown that chloroplast translation is reduced in young *pp7l* mutant seedlings (Xu et al., 2019). Therefore, we conducted a study on the impact of drought on the translation of chloroplast transcripts using *in vivo* labeling, which allows for measuring *de novo* protein synthesis. We monitored the synthesis of plastid-encoded proteins through pulse labeling of both WT and *pp7l-1* mutant plants in the presence of cycloheximide, which blocks the translation of nuclear-encoded proteins. We carried out this monitoring before and during drought stress, as well as three days after re-watering. After 30 minutes of pulse labeling, *pp7l-1* exhibited a higher amount of newly-formed RbcL, D1, and D2 proteins compared to WT under conditions of drought. However, the synthesis of these proteins was reduced in *pp7l-1* compared to WT after 3 days of re-watering (**Figure 1G**). Interestingly, PP7L is localized to the nucleus and the cytosol, but not to chloroplasts (Xu et al., 2019; de Luxán-Hernández et al., 2020), and how PP7L contributes to chloroplast translation and development remains unresolved (Xu et al., 2019). Therefore, to test whether mutants directly impaired in plastid gene expression are also and generally more drought tolerant, we examined plants with mutations in *PROLYL-tRNA SYNTHETASE1* (*PRORS1*) and SIGMA6 (*SIG6*). The *prors1* and *sig6* mutants are impaired in organellar translation (Pesaresi et al., 2006) and transcription (Ishizaki et al., 2005), respectively. After prolonged drought stress, *SIG6* mRNA levels accumulated in *pp7l* compared to WT, while the effect on *PRORS1* transcript levels was milder (**Figure 1H**). When WT and mutant plants were subjected to drought stress, WT plants wilted after 16 days, while the *prors1* and *sig6* mutants wilted after 20 and 24 days, respectively (**Figure 1I**). However, the *pp7l* mutant remained healthy throughout the whole period.

We conclude that – in contrast to the WT – chloroplast development in the *pp7l* mutant is positively influenced by drought stress. Additionally, a disturbance in chloroplast gene expression does not generally lead to drought tolerance as observed when PP7L is lacking.

### 3.3 Drought stress and its impact on the *pp7l* chloroplast (post)transcriptome

Prompted by the long survival and recovery of Fv/Fm in the *pp7l* mutant under drought stress which might be interesting for both organellar and nuclear RNA expression patterns, RNA sequencing was performed in a way that allowed to detect nuclear as well as organellar transcripts. However, it is important to note that the identification of tRNAs poses a challenge. Because they are small in size and have numerous modifications, they are difficult to amplify by both the RNA-Seq method employed in this publication and conventional mRNA-Seq protocols. RNA was extracted from 3-week-old *pp7l* plants grown under well-watered conditions (0 days post-treatment, or 0d DS) and following water deprivation for 6, 9, 12, and 15 days (referred to as 6d_DS, 9d_DS, 12d_DS, and 15d_DS, respectively) using previously described methods (Kim et al., 2017). These plants were grown in conjunction with previously characterized WT plants (Xu et al., 2023). The study employed several plant batches, resulting in three biological replicates for every time point. Each sample was sequenced to a depth of approximately 25 million paired-end (150 bp) reads, as detailed in **Supplementary Table 1**.

**Figure 2:**
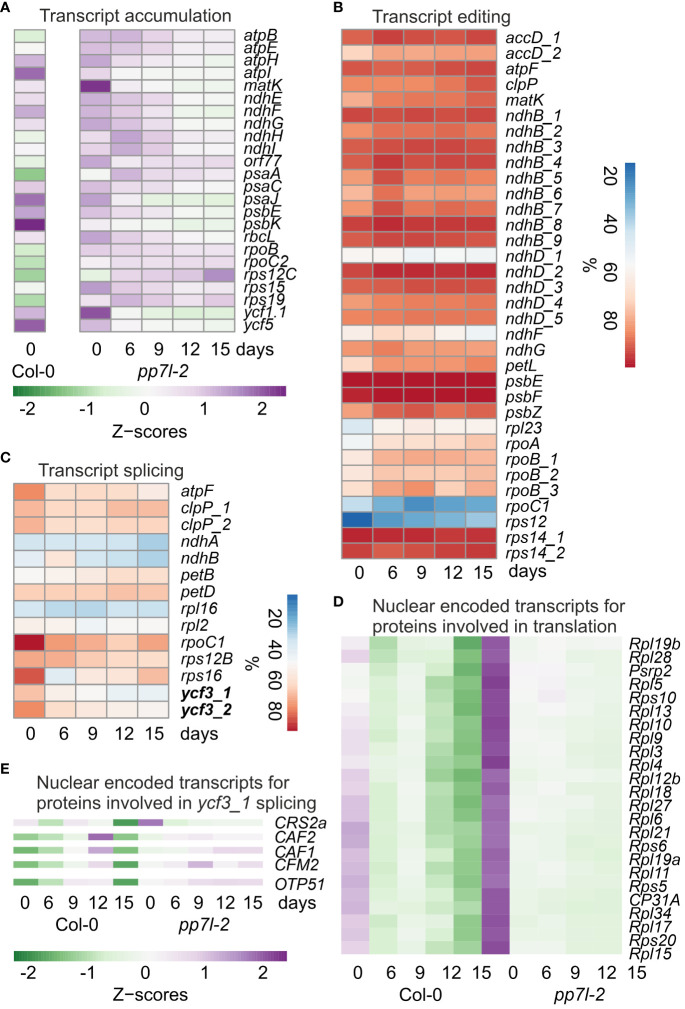
Impact of drought stress on chloroplast transcript accumulation, editing and splicing. **(A)** A heatmap that illustrates the accumulation of selected chloroplast transcripts (Z-scores) during the drought time-course is provided. The transition from low to high expression is shown by the shift in color from green to purple. It is important to note that Z-scores are calculated for each individual transcript across the entire time course. The WT data were obtained from Xu et al. (2023). The percentages of editing **(B)** and splicing **(C)** events during the drought time-course are presented. The accumulation of nuclear-encoded transcripts for proteins related to chloroplast translation **(D)** and *ycf3_1* splicing **(E)** during the drought time-course is depicted in a heatmap.

Because of the strong effect of drought stress on the Fv/Fm phenotype of *pp7l* plants, we first investigated accumulation of plastid transcripts under drought stress. A gene was classified as differentially expressed (DEG) if it exhibited a ≥ 2-fold linear change (an absolute log_2_ fold change ≥1) in expression in at least one comparison group (adjusted *P* < 0.05; **Supplementary Table 2**). To illustrate expression changes, heatmaps of Z-means were generated for transcripts that showed expression changes in at least one condition examined (**Figure 2A**). First, expression changes between *pp7l* and WT plants under control growth conditions (time point 0) were analyzed. Of the 71 transcripts above the threshold level and encoding proteins, five transcripts were elevated and only one reduced which was that of *psbK* encoding PSII reaction center protein K. Notably, in 4-day-old *pp7l* seedlings grown under well-watered conditions, plastid transcripts also generally accumulated to higher levels than those observed in wild-type controls (Xu et al., 2019). In addition, three, four, 18, and 17 transcripts were at least 2-fold reduced (compared to the 0d_DS sample) in *pp7l* plants after 6, 9, 12 and 15 days of drought, respectively, while no transcript was induced. However, the relatively constant – and transiently even higher, although not 2-fold – expression of genes encoding the PSII reaction center proteins PsbA, PsbC, and PsbD in *pp7l* plants throughout drought stress is noteworthy (**Supplementary Table 2**). The most affected gene that was already down-regulated after 6 days codes for MatK, the only chloroplast-localized splicing factor. MatK is involved in splicing of several *tRNA*s, *atpF*, *rpl2* and *rps12–2* (de Longevialle et al., 2010). Genes whose transcripts were reduced during the later time points code for example for the F, G, and H subunits of the NAD(P)H dehydrogenase complex, the PSII subunits K and E, the PSI subunits J and C and ribosomal protein S15.

To investigate post-transcriptional alterations in plastid transcripts, we calculated editing and splicing efficiencies. It was observed that editing efficiency was not significantly reduced, and, in some instances, even slightly improved (**Figure 2B**). However, splicing efficiency was reduced under drought stress for 5 out of 14 transcripts (**Figure 2C**). Interestingly, this is in contrast to the WT, where splicing efficiency was not significantly decreased after 15 d of drought stress, but in some cases was slightly increased (Xu et al., 2023). With the exception of MatK, all splicing and editing factors are encoded in the nuclear genome, and chloroplast ribosomes are a mosaic of proteins encoded by both the plastid and the nucleus. To investigate a putative correlation between nuclear gene expression of chloroplast splicing factors and the respective splicing events, transcript accumulation of genes encoding proteins involved in chloroplast gene expression were determined. Here, also transcript accumulation of WT plants under drought was incorporated. Plotting their Z-scores and sorting the transcripts into clusters according to their expression performance under drought, resulted in two clusters (**Supplementary Figure 2**). One cluster showed a transient up-regulation of transcripts, and a final down-regulation after 15 d in the WT, while these transcripts did not change or were slightly up-regulated in *pp7l*. In the other cluster transcript accumulation was higher in *pp7l* under well-watered conditions than in the WT, but transcripts decreased under drought in both WT and *pp7l* (**Figure 2D**, **Supplementary Figure 2**). This cluster contains genes encoding mainly ribosomal proteins, which is intuitively in contrast to the improved chloroplast translation in *pp7l* under drought stress (see **Figure 1G**). In addition, there appears to be no simple correlation between the accumulation of nuclear-encoded chloroplast transcript maturation factors and chloroplast post-transcriptomic changes. This is also due to complex interactions of splicing factors. For example, in land plants, intron 1 of *ycf3* (*ycf3_1*) is spliced by at least CRM Family Member 2 (CFM2) and RNA splicing factor CRS2 together with CRS2-associated factors CAF1 and 2 (de Longevialle et al., 2010), but only *CRS2* transcripts were decreased under drought (**Figure 2E**). Intron 2 of *ycf3* is spliced by ORGANELLAR TRANSCRIPT PROCESSING 51 (OTP51) (de Longevialle et al., 2010), whose transcripts are slightly enhanced under drought (**Figure 2E**), whereas splicing of *ycf3_2* is reduced (**Figure 2C**).

In conclusion, there is no simple correlation between mRNA levels of nuclear-encoded genes affecting plastid gene expression and plastid (post)-transcriptome changes under drought.

### 3.4 Behavior of cell wall mutants under drought conditions

**Figure 3:**
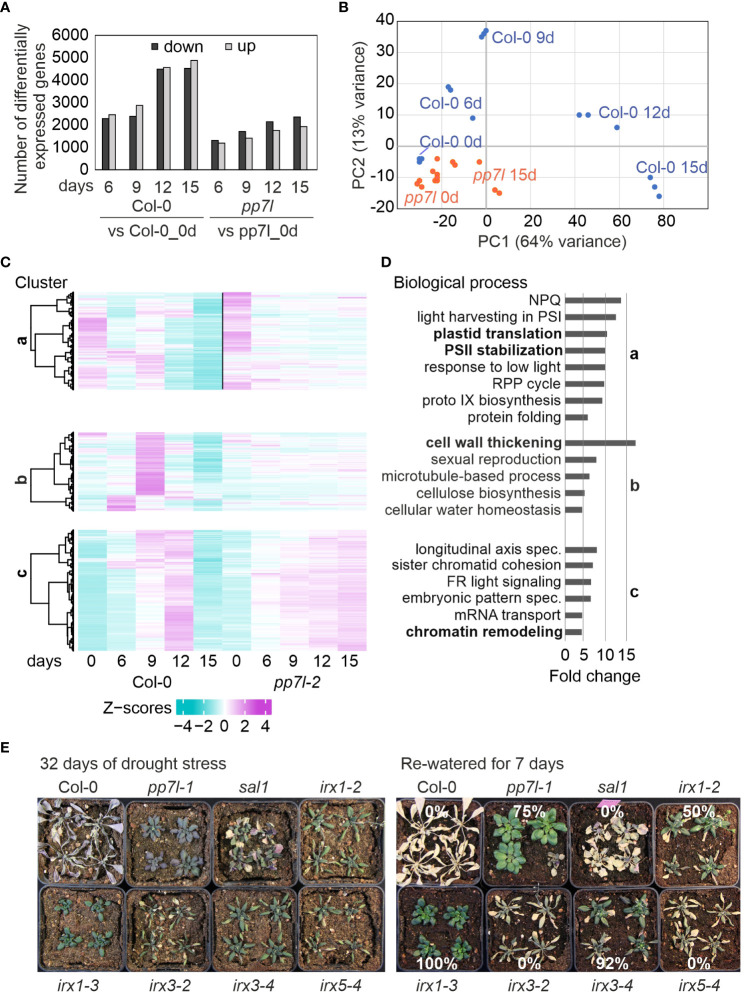
Overview and Gene Ontology (GO) analysis regarding gene expression alterations during drought conditions. **(A)** The number of differentially expressed (DE) genes (with an absolute log_2_ fold change greater than or equal to 1 and an adjusted *P*-value of less than 0.01 in at least one contrast group) in WT and *pp7l* plants exposed to drought compared to their respective controls (0 days) is presented. **(B)** RNA-seq data-based PCA plot demonstrates the variance between genotypes and time-points. **(C)** We employed hierarchical clustering with the Euclidean distance and ward.D clustering algorithm to partition the DE genes into clusters. **(D)** Graphs indicating non-redundant GO term enrichment for the biological process category were presented according to DAVID (Huang da et al., 2009). Only those GO terms with a ≥5-fold change and a Benjamini-corrected *P*-value of < 0.05 are displayed. LHC, light-harvesting complex; NPQ, nonphotochemical quenching; RPP, reductive pentose-phosphate; PS, photosystem. **(E)** WT and the different genotypes were grown for 3 weeks under control conditions, water was withheld for 32 days, and then plants were re-watered for 7 days.

In addition, we compared the overall nuclear gene expression behavior of *pp7l* under drought with that of WT plants using the RNA-Seq data we generated in this and our previous study (Xu et al., 2023). A gene was categorized as a DEG if its expression displayed an absolute log_2_ change ≥1 in at least one comparison group, and we applied a more stringent adjusted *P* of < 0.01. Slightly more genes were down- (54.4%) than up-regulated in *pp7l*, but the differences in gene expression increased only moderately when compared to the previously generated WT dataset (**Figure 3A**; **Supplementary Table 3**). This is further demonstrated by a PCA (principal component analysis) conducted on gene expression data from the samples, which showed that drought (64% of variance) was the main driver of gene expression in the WT, but not in *pp7l* (**Figure 3B**).

The kinetic data were further analyzed by sorting the transcript changes related to *pp7l* into the nine clusters previously identified in the WT drought setup (Xu et al., 2023), and three clusters caught our attention. Cluster a contains the genes that were strongly down-regulated in the WT at 12d_DS and 15d_DS, but these genes are only moderately repressed in *pp7l*. GO (Gene Ontology) analysis of this cluster revealed enriched GOs related to plastid translation, chlorophyll biosynthesis, and photosynthesis-related processes within the BP (biological process) category (**Figures 3C, D**). This underscores the greater susceptibility of WT plants to drought stress.

Genes associated with the GO categories “cell wall thickening” and “cellular water homeostasis” (enriched more than 15-fold) in cluster b are induced specifically in WT plants after 6 or 9 days of drought stress, and decline under progressive drought, but are only slightly changed in *pp7l*. It was previously found that disruption of *CELLULOSE SYNTHASE GENE 8* (*CESA8*; *IRREGULAR XYLEM 1*, *IRX1*) enhances drought and osmotic stress tolerance in Arabidopsis (Chen et al., 2005). Inspection of the cell wall thickening genes in cluster b showed that *IRX1* was indeed included, as well as *IRX3*, *IRX5* and *IRX9*. IRX1, 3 and 5 are involved in cellulose synthesis of the secondary cell wall (Taylor et al., 2003), and it was noted before that a defect in IRX1, IRX5 (Hernandez-Blanco et al., 2007) or IRX3 (Xu et al., 2020a), results in the up-regulation of ABA-responsive genes. However, it is still unclear how the distinct CESA/IRX proteins are arranged within the CSC (Cellulose Synthase Complex). CESA7/IRX3 has been found to hold a highly limited position within the CSC. In contrast, CESA8/IRX1 appears to have low class specificity (Kumar et al., 2017). To observe the drought response of *irx1* mutants under our drought conditions, to confirm that other *irx1* mutant alleles are also drought tolerant, and to clarify whether disruption of *IRX3* or *IRX5* can confer drought tolerance or that of IRX3 can even lead to higher drought tolerance, we examined previously identified *irx1–2*, *irx1–3*, *irx3–2*, *irx3–4*, and *irx5–4* mutants (Xu et al., 2020a) in comparison to *pp7l* and *sal1* mutants when water was withheld for 32 days and then re-watered for 7 days. After re-watering, WT, *sal1*, *irx3–2*, and *irx5–4* plants died (**Figure 3E**). However, *irx1–2*, *pp7l*, *irx3–4*, and *irx1–3* plants had survival rates of 50%, 75%, 92%, and 100%, respectively. Of note is that this variation among alleles was already previously observed when studying the performance of cell wall mutants under conditions that restrict chloroplast translation (Xu et al., 2020a).

In summary, drought stress triggers a significant reprogramming of transcript abundances for cell wall proteins. However, of the cell wall mutants investigated, specifically IRX1 deficiency in *irx1–3* reliably confers drought tolerance.

### 3.5 The double mutant *pp7l hda6* is extremely drought resistant

**Figure 4:**
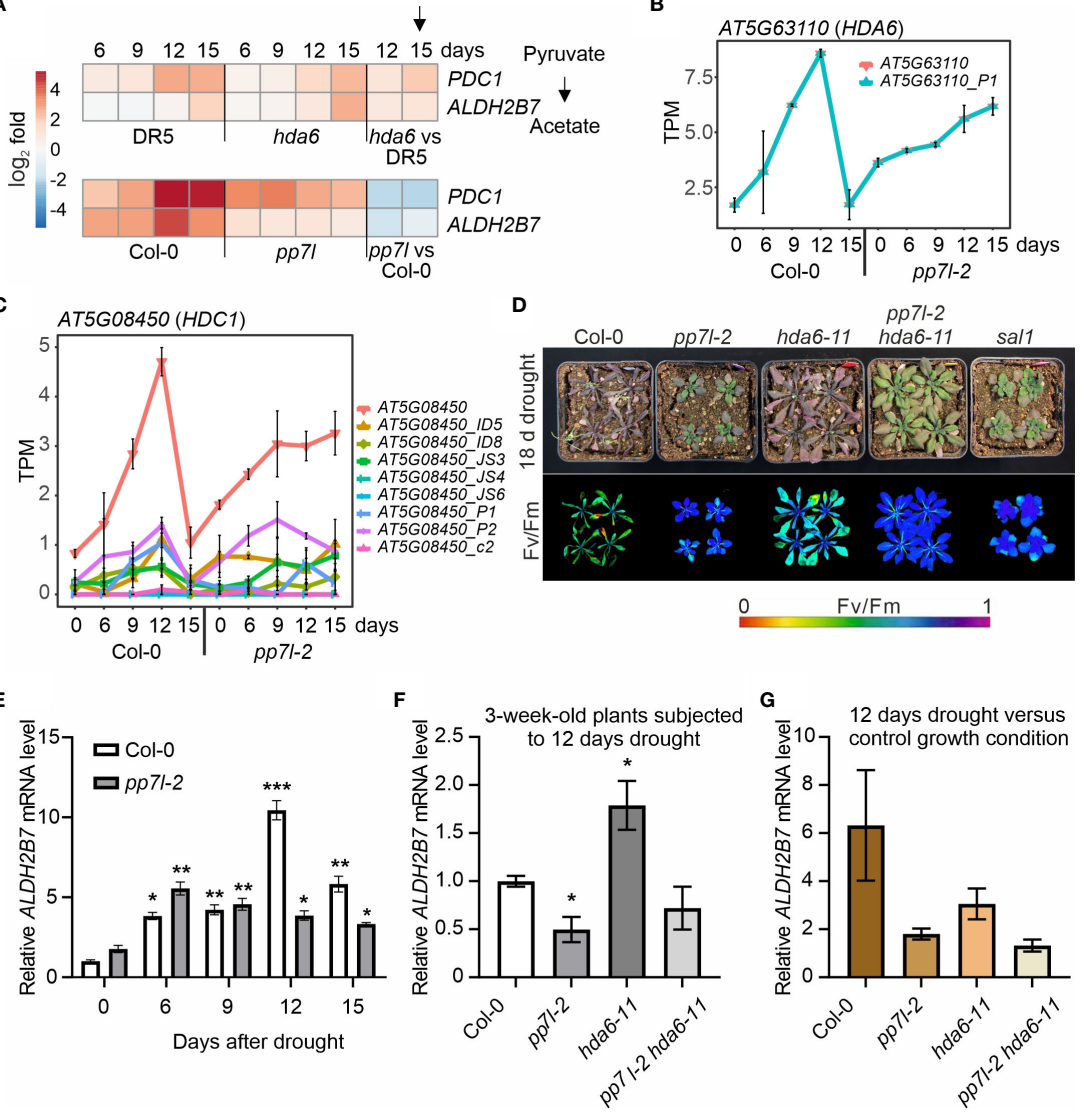
The *pp7l hda6* mutant is extremely drought resistant. **(A)** Heatmap illustrating the log_2_ fold changes compared to time-point 0 days of pyruvate decarboxylase *PDC1* and acetaldehyde dehydrogenase *ALDH2B7* transcripts in the experiment conducted by Kim et al. (2017) (upper panel) and in this study (lower panel). **(B, C)** Expression profiles of *HDA6* **(B)** and *HDC1* **(C)** are presented at both the level over the whole gene and detected transcript isoforms. PS represents the percentage of spliced expressed transcripts, and TPM the transcripts per million reads. **(D)** WT and mutant plants were grown separately in pots within the same container, and water was withheld from 3-week-old plants for 18 days. **(E)** qRT-PCR analysis of WT and *pp7l-2* mutant plants under control growth conditions and subjected to drought stress for 6, 9, 12 and 15 days. qRT-PCR was performed with primers specific for *ALDH2B7* and *AT3G58500* encoding PROTEIN PHOSPHATASE 2A-4. The results were normalized with respect to the expression level of *PP2A-4*. Bars indicate standard deviations. Statistically significant differences are indicated (*, *P* < 0.05; **, *P* < 0.01; ***, *P* < 0.001). **(F)** qRT-PCR analysis of WT and mutant plants under control growth conditions and subjected to drought stress for 12 days was performed as in panel **(E)**. **(G)** To account for the differences in *ALDH2B7* expression among the different genotypes observed in the absence of drought stress, the ratio of the *ALDH2B7* expression level in plants subjected to 12 days of drought stress to that in plants grown under normal growth conditions was calculated.

As discussed in Xu et al. (2023), there is no model that can meet all the requirements for drought studies that would mimic natural conditions in the field. This raises the need for more community standardization of drought stress experiments (VanBuren et al., 2024). A setup, in which water is withheld from plants until wilting is observed (Harb and Pereira 2010), is accessible to all laboratories and appears to be robust as previously shown (Xu et al., 2023). Comparison of transcriptome changes generated from two different studies using even two different methodologies (Kim et al., 2017; Xu et al., 2023) showed that the transcriptome changes evoked by 12 and 15 days of drought stress were similar. While Xu et al. (2023) and this study use Col-0 as the wild type, Kim et al. (2017) employed the *DR5*::*GUS* (short: DR5, in the Col-0 background) line as a control. This line was originally used to screen for *auxin gene expression* (*axe*) mutants, and *axe1–5* was identified, in which *HDA6* is mutated (Murfett et al., 2001). Later, Kim et al. (2017) identified acetate as a driver of drought resistance, based on the observation that, under prolonged drought stress, larger amounts of *PDC1* and *ALDH2B7* transcripts accumulated in the drought-tolerant *histone deacetylase 6* (*hda6; axe1–5*) mutant than in the control line DR5 (Kim et al., 2017; **Figure 4A**). Both of these genes code for enzymes in the acetate biosynthesis pathway. In our setup, the degree of induction of *PDC1* and *ALDH2B7* was even higher in our control WT than in DR5 which was used by Kim et al. (2017). In drought-exposed *pp7l* plants, these transcripts were also induced, but at levels lower than that seen in WT plants (**Figure 4A**). This expression behavior contrasts sharply with the effect of the *hda6* mutation compared to the DR5 control used by Kim et al. (2017) (**Figure 4A**), suggesting that different mechanisms underlie the drought tolerance of *hda6* and *pp7l* mutants, respectively. Therefore, we searched for genes that behaved in our *pp7l*/WT data similar as seen for *PDC1* and *ALDH2B7* in the *hda6*/DR5 dataset. The third cluster (cluster c) caught our attention. This cluster comprises genes whose transcript levels transiently increased in our WT control and dropped after prolonged drought, but remained up-regulated in *pp7l* from 9 days of drought stress on. Strikingly, the GO term “chromatin remodeling” is enriched in this cluster, and *HDA6* itself shows such an expression profile, together with *HISTONE DEACETYLATION COMPLEX 1* (*HDC1*; **Figures 4B, C**). Note here the differential accumulation of *HDC1* isoforms (**Figure 4C**). HDC1 works together with HDA6 and HDA19 to facilitate the deacetylation of histones (Perrella et al., 2013). Moreover, levels of both *HDA6* and *HDC1* mRNAs were already elevated in *pp7l* relative to the WT under standard growth conditions. This finding was unexpected, because theoretically, this would imply that HDA6 and its interactor are required to *withstand* drought. Hence, introduction of a *HDA6* mutation into *pp7l* would be expected to abrogate *pp7l*’s drought-tolerant phenotype, although the *hda6* single mutant is drought tolerant. In addition, PP7L and HDA6 regulate gene expression and repress TEs. However, the molecular function of PP7L remains unclear (see Introduction; de Luxán-Hernández et al., 2020; Nicolau et al., 2020). We reasoned that combining HDA6 and PP7L mutations may help identify the (epigenetic) process in which PP7L is involved. To test our assumption, we created *pp7l hda6* double mutants (**Supplementary Figure 3**). To be independent of the DR5 line, we used *hda6–7* and *hda6–11* mutants in our experiments, which are in the Col-0 background. When water was withheld from 3-week-old WT, *hda6*, *pp7l*, *pp7l hda6* and – as a control – *sal1* plants, the WT plants had begun to die after 18 days, and their Fv/Fm values were very low (**Figure 4D**). In contrast, the younger leaves of *hda6*, *sal1* and *pp7l* plants were still able to photosynthesize and did not wilt, corroborating our findings and earlier reports of the drought resistant *hda6* (Kim et al., 2017) and *sal1* (Wilson et al., 2009) phenotypes. Surprisingly and remarkably, the *pp7l-1 hda6–7* and *pp7l-2 hda6–11* double mutant plants outperformed all other mutants, displaying the highest photosynthetic capacity, low anthocyanin accumulation and no signs of wilting (**Figure 4D**; **Supplementary Figure 3**). Thus, the *pp7l hda6* double mutant alleles displayed additive phenotypes compared to the single mutants, indicating distinct mechanisms of action for PP7L and HDA6. Moreover, because of the counterintuitive result concerning the drought phenotype, we attempted to determine *ALDH2B7* mRNA levels in WT, *pp7l*, *hda6* and *pp7l hda6* plants under drought conditions. To this end, the drought time course experiment was repeated with WT, *pp7l-2*, *hda6–11* and *pp7l-2 hda6–11* plants. Determination of *ALDH2B7* mRNA levels by quantitative reverse transcriptase-polymerase chain reaction (qRT-PCR) analysis on RNAs extracted from WT and *pp7l-2* confirmed the RNA-Seq data generated in our first time course (see **Figure 4A**), and again demonstrated the robustness of the drought experiment by water withholding (Xu et al., 2023). While *ALDH2B7* mRNA levels raised in both WT and *pp7l-2* after water was withheld from 3-week-old plants for 6, 9, 12, and 15 days, this increase was much more pronounced in WT (**Figure 4E**). Because *ALDH2B7* transcript levels peaked after 12 days of drought stress in WT and the difference between WT and *pp7l-2* was most pronounced, we chose this time point to also detect *ALDH2B7* mRNA levels in *hda6–11* and *pp7l-2 hda6–11* mutant plants. After 12 days of drought treatment, *ALDH2B7* mRNA levels were 2-fold elevated in *hda6* plants compared to the WT (**Figure 4F**), corroborating the results of the transcriptome data shown in **Figure 4A**, but they were not significantly different in the *pp7l hda6* double mutant (**Figure 4F**). In addition, *ALDH2B7* mRNA levels were already increased in *hda6* and *pp7l hda6* plants under control conditions. To account for this difference the ratio of the *ALDH2B7* expression level in plants subjected to 12 days of drought stress to that in plants grown under normal growth conditions was calculated (**Figure 4G**). Although the 3-fold increase in *ALDH2B7* mRNA levels in the *hda6* mutant was consistent with the transcriptome data shown in **Figure 4A**, the induction was not as strong as in the WT (6-fold induction), and the *pp7l hda6* mutant behaved like the *pp7l* single mutant (**Figure 4F**). Therefore, under our experimental conditions, acetate may not be the primary driver for the drought resistance of the *pp7l*, *hda6*, and *pp7l hda6* mutants. This result also reflects the complexity of the salt and drought resistance of the *hda6* and *hda19* mutants described in the Introduction.

The molecular mechanism leading to the extremely drought-resistant phenotype of the *pp7l hda6* mutant was not resolved yet. But notably, the dwarf phenotype of *pp7l* was suppressed in the *pp7l hda6* double mutant. Consequently, a dwarf effect on the drought-tolerant phenotype of this genotype under our experimental conditions can be excluded.

In conclusion, these results show that both PP7L and HDA6 constrain drought tolerance, and that loss of both proteins results in an extremely drought-resistant mutant in which growth penalty of *pp7l* has been released. Future research may consider the involvement of multiple loci and factors involved in stress resistance and investigate combinations of them to create even more resistant plants.

## Data availability statement

The data presented in the study are deposited in the Gene Expression Omnibus repository (Edgar et al., 2002), accession number GSE202931.

## Author contributions

DX: Conceptualization, Formal analysis, Investigation, Validation, Visualization, Writing – review & editing. DL: Funding acquisition, Resources, Writing – review & editing. TK: Conceptualization, Data curation, Formal analysis, Funding acquisition, Investigation, Methodology, Project administration, Resources, Supervision, Validation, Visualization, Writing – original draft, Writing – review & editing.
