# Supplementary material for: Identification of a highly drought-resistant pp7l hda6 mutant
Source: Front Plant Sci. 2024 Jun 3;15:1341576. doi: 10.3389/fpls.2024.1341576 (PMC11180769; doi:10.3389/fpls.2024.1341576)
Supplement: Supplementary file 1 [file DataSheet_1.pdf]

*Supplementary Material*

# Identification of a highly drought-resistant *pp7l hda6* mutant

**Duorong Xu[1], Dario Leister[1], and Tatjana Kleine[1,*]**

*** Correspondence:**
Dr. Tatjana Kleine

Ludwig-Maximilians-Universität München (LMU)
Department Biologie I, Botanik; Großhaderner Str. 2, D-82152 Planegg-Martinsried
Phone: +49-89/2180 74554; Fax: +49-89/2180 74599; E-mail: tatjana.kleine@lmu.de

## 1 Supplementary Figures and Tables

### 1.1 Supplementary Figures

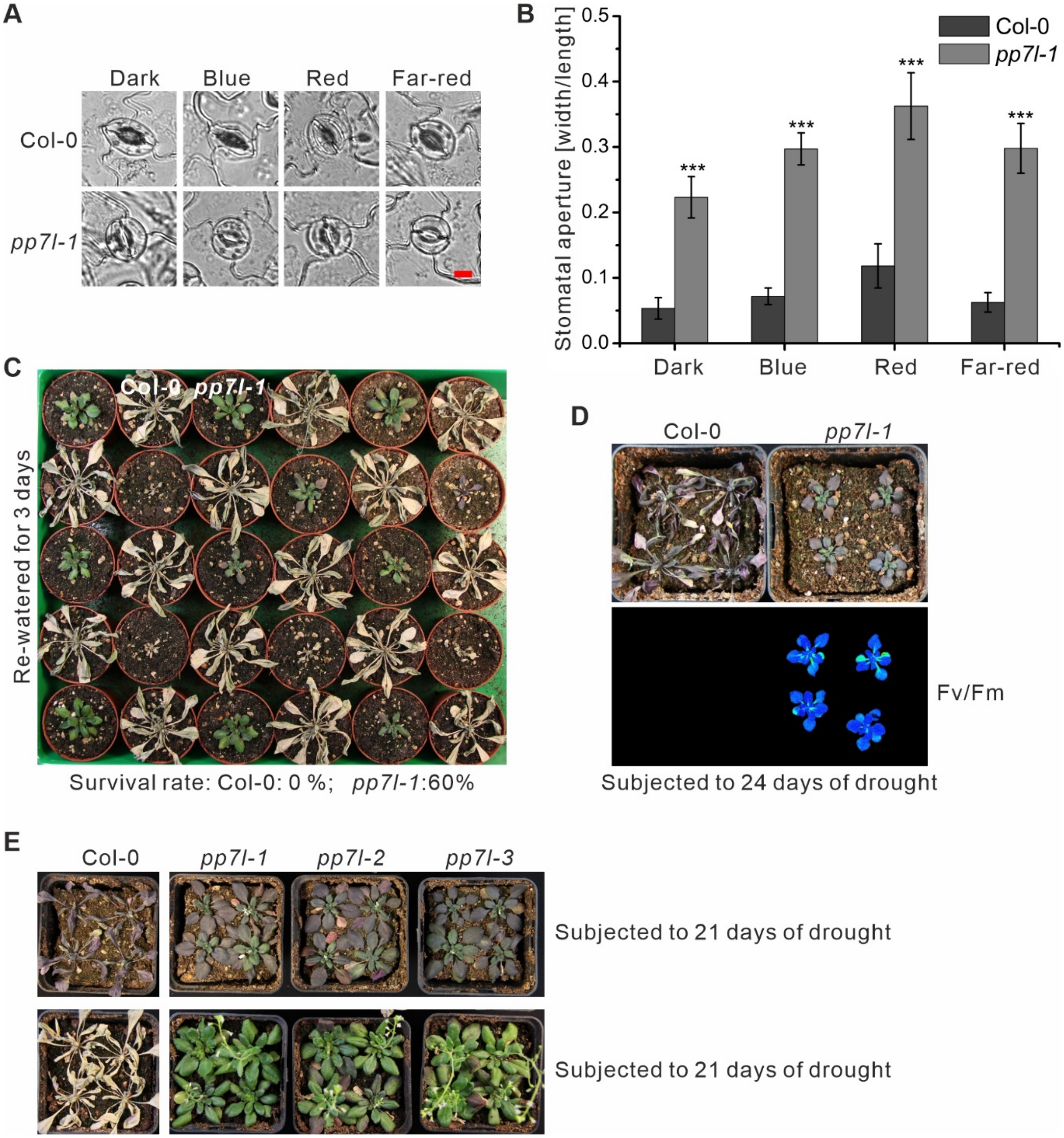


**Supplementary Figure 1.** The *pp7l* mutant can survive long periods of drought stress and behavior of stomata of WT (Col-0) and *pp7l-1* plants in darkness, under different lighting conditions and under drought. **(A)** The micrographs show stomatal opening in leaves of WT and *pp7l* mutant plants grown in the dark, or subjected to 30 µmol $m^{-2}$ $s^{-1}$ blue light, 50 µmol $m^{-2}$ $s^{-1}$ red light or 50 µmol $m^{-2}$ $s^{-1}$ far-red light. Bar =10 µm. **(B)** Analysis of stomatal aperture morphology in leaves of WT and *pp7l-1* grown as in in (A). Student's t test, ***$P$ < 0.001. **(C)** Three-week-old plants were subjected to drought stress by withholding water for 18 days and were then re-watered for 3 days. **(D)** Phenotypes and Imaging PAM pictures of three-week-old plants that were subjected to drought stress by withholding water for 24 days. **(E)** Phenotypes of four-week-old plants that were subjected to drought stress by water deprivation for 21 days (top panel) and then re-watered for 7 days (bottom panel).

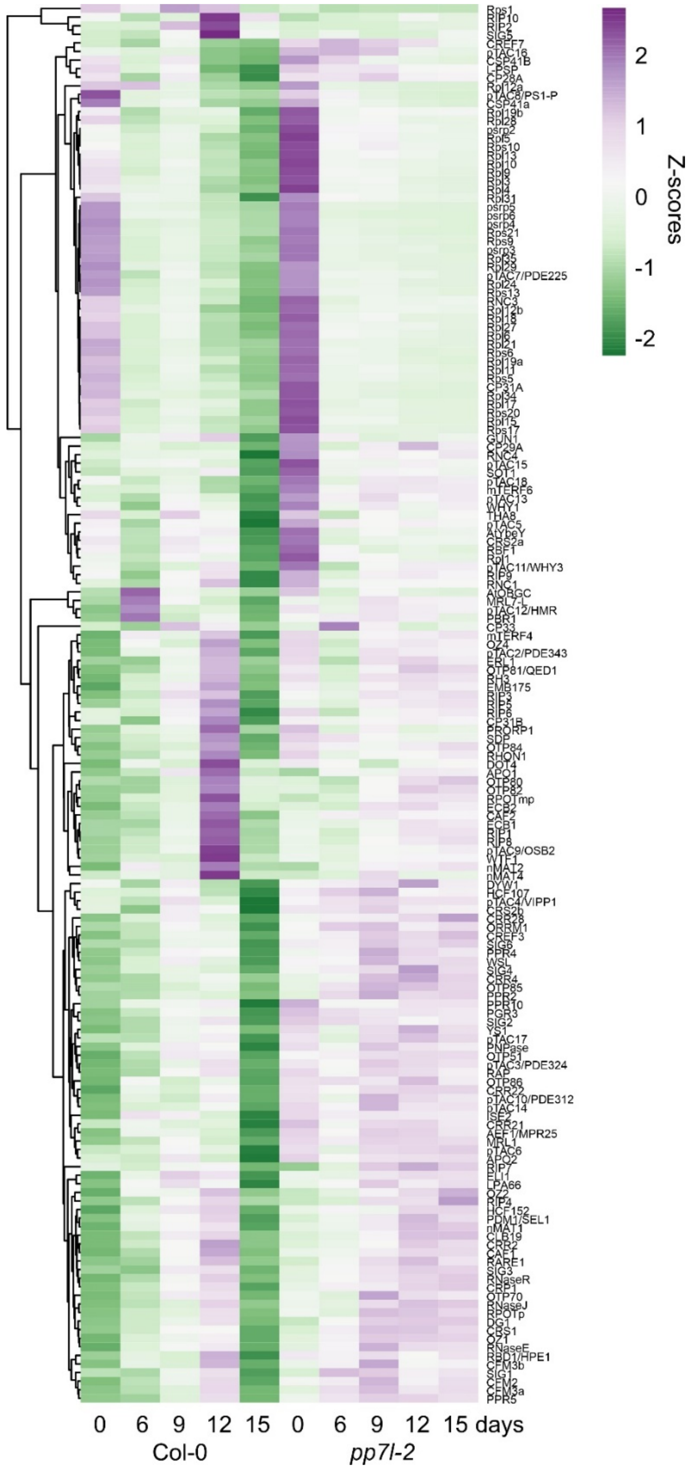


**Supplementary Figure 2.** Hierarchical clustering of transcript accumulation of nuclear genes encoding proteins involved in chloroplast gene expression. Plotting their Z-scores and sorting the transcripts into clusters according to their expression performance under drought, resulted in two clusters.

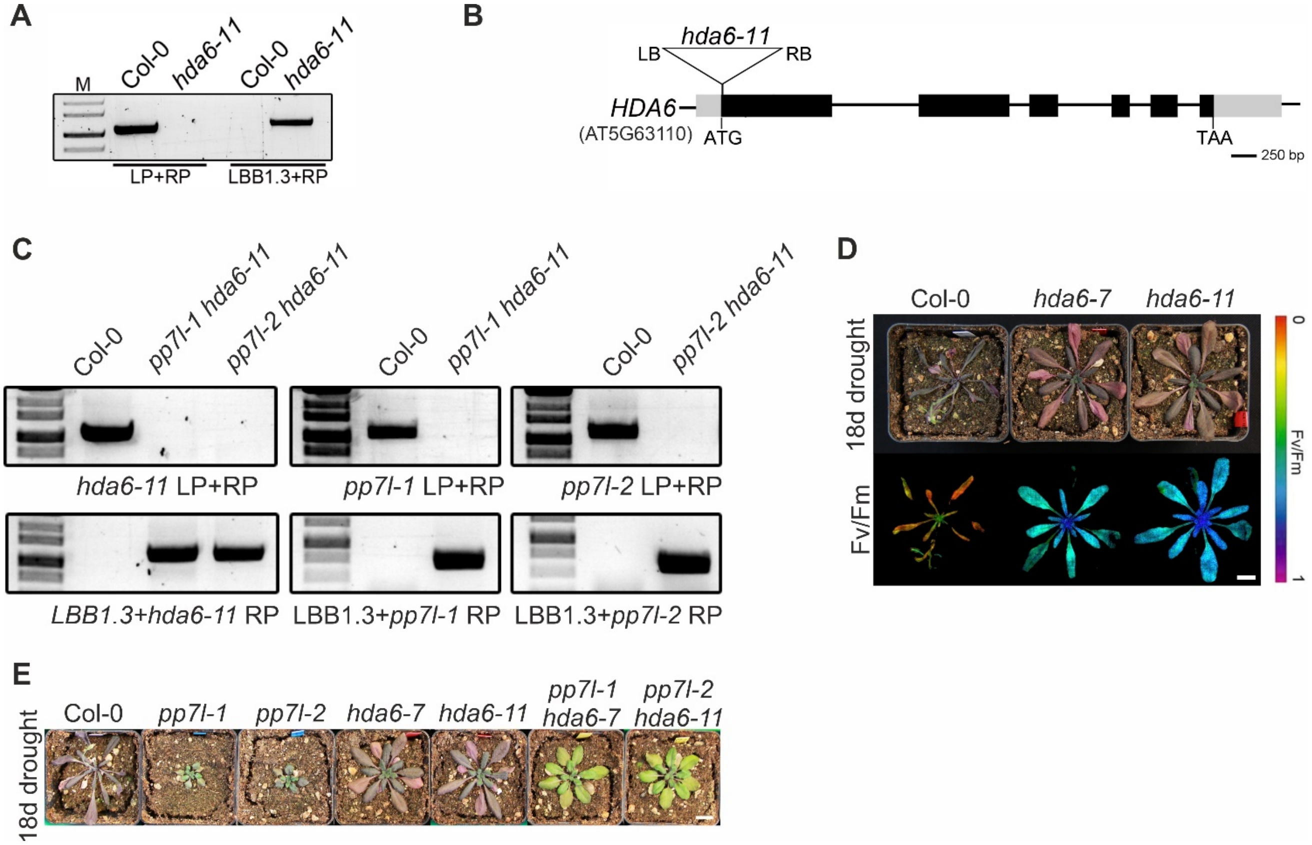


**Supplementary Figure 3.** Identification of the *pp7l-1 hda6-11* T-DNA insertion mutant. **(A)** Confirmation and identification of homozygous T-DNA insertion in the *hda6-11* mutant line. The gene-specific left and right primers (LP and RP) were used for amplification of sequences around the T-DNA insertion, and RP was used together with the T-DNA left border primer (LBB1.3) for verification of the T-DNA insertion. (**B**) Schematic representation and T-DNA tagging of the *HDA6* (*AT5G63110*) locus. Exons (black boxes), introns (black lines) and the 5' and 3' UTRs (grey boxes) are shown. Numbers are given relative to the start codon ATG. Locations and orientation of T-DNA insertions are indicated, as deduced from RP + LB PCR products shown in (A)**,** which were subsequently sequenced. Note that the insertions are not drawn to scale. **(C)** Genotyping of *pp7l-1 hda6-11* and *pp7l-2 hda6-11* mutants. PCR was performed with genomic DNA prepared from the respective plants, and PCR was performed with primer sets indicated below the gel picture. **(D)** Drought tolerance of two different *hda6* mutants compared with the WT. Photographs were taken after 18 days of drought exposure. Bar = 1 cm. **(E)** Drought tolerance of *pp7l-1 hda6-11 and pp7l-2 hda6-11* double mutants compared with the single mutants and the WT. Photographs were taken after 18 days of drought exposure. Bar = 1 cm.

## 1.2 Supplementary Tables

**In separate files:**

**Supplementary Table 1.** Sequencing depth of RNA-Seq experiments. RNA sequencing (RNA-Seq) was performed with RNA isolated from 3-week-old *pp7l* plants grown under optimal conditions

(time-point 0 days; 0d) and after water had been withheld for 6, 9, 12 and 15 days, respectively (6d, 9d, 12d and 15d).

**Supplementary Table 2.** Abundance of chloroplast transcripts. $Log_2$ fold changes of transcripts encoded by the chloroplast genome. Note that analysis of tRNAs was excluded.

**Supplementary Table 3.** Genes differentially expressed under drought stress. RNA sequencing (RNA-Seq) was performed with RNA isolated from 3-week-old WT (Col-0; Col) (Xu et al., 2023) and *pp7l* (this publication) plants grown under optimal conditions (time-point 0 days; 0d) and after water had been withheld for 6, 9, 12 and 15 days, respectively (6d, 9d, 12d and 15d).
